# Infrapyloric Lymph Node Dissection Technique in Robotic Gastrectomy With Recognition of the Embryological Fusion Plane

**DOI:** 10.7759/cureus.99400

**Published:** 2025-12-16

**Authors:** Ippei Yamana, Takahisa Fujikawa, Toshifumi Watanabe, Yuichiro Kawamura, Suguru Hasegawa

**Affiliations:** 1 Surgery, Kokura Memorial Hospital, Kitakyushu, JPN; 2 Gastroenterological Surgery, Fukuoka University Hospital, Fukuoka, JPN

**Keywords:** gastric cancer surgery, lymph node dissection, peripancreatic lymph node, robotic gastrectomy, robotic surgical procedures

## Abstract

Robotic gastrectomy (RG) for gastric cancer has gained popularity in recent years, facilitating more intricate surgical procedures. However, the technical complexity of infra-pyloric lymph node dissection in RG arises from the complex anatomy of the region. Embryologically, the pancreatic head is formed by the fusion of the ventral and dorsal pancreatic primordia during development, and recognizing the embryological fusion plane (EFP) may help prevent misleading results during infra-pyloric lymph node dissection. This report presents an operative technique that emphasizes the importance of recognizing the EFP.

## Introduction

The limitations of traditional laparoscopic gastrectomy have been overcome by robotic gastrectomy (RG) using the da Vinci Surgical System (Intuitive Surgical), which has made surgery safer and more precise [1]. Safe and accurate lymph node dissection is important in RG for gastric cancer. Infrapyloric lymph node dissection is required for both total and distal gastrectomy. However, due to the complex anatomy of the pancreatic head, this is the most challenging site for lymph node dissection in gastric cancer surgery. In addition, care must be taken to avoid complications such as pancreatic fistula and bleeding.

It is essential to understand the anatomy of the pancreatic head, which is formed by the rotational fusion of the ventral and dorsal pancreatic primordia. The plane between the dorsal pancreas and the ventral pancreas is called the embryological fusion plane (EFP) [2-7]. The 3D magnification effect of the robotic system enables the recognition of the EFP. Situated along this EFP is the right gastroepiploic artery, which serves as an important landmark during infrapyloric lymph node dissection.

So far, few technical methods for infrapyloric lymph node dissection during RG have been reported [8,9]. Therefore, we present a technique for infrapyloric lymph node dissection in RG, emphasizing the recognition of the EFP.

## Technical report

Anatomy of the ventral and dorsal pancreas

**Figure 1:**
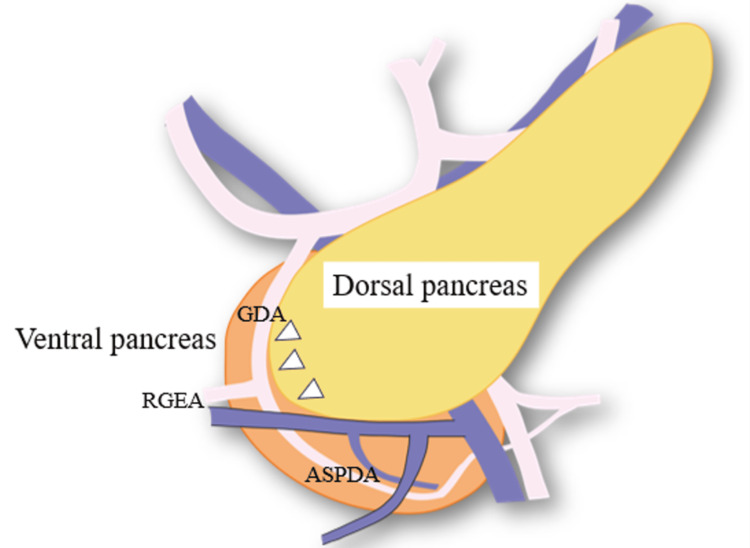
Illustration of the partition separating the ventral and dorsal pancreases. The plane between the dorsal pancreas and the ventral pancreas is called the embryological fusion plane (EFP). Arrows: EFP; Orange: Ventral pancreas; Yellow: Dorsal pancreas. GDA: Gastroduodenal artery; RGEA: Right gastroepiploic artery; ASPDA: Anterior superior pancreatic duodenal artery. Image credit: Yuichiro Kawamura.

The head of the pancreas is embryologically composed of the ventral and dorsal pancreatic primordia. As the stomach and duodenum rotate, the ventral pancreatic bud shifts to the dorsal side and merges with the dorsal bud, resulting in the formation of a single pancreas. The dorsal pancreatic bud develops into the ventral section of the head, neck, body, and tail of the pancreas, while the ventral bud provides the dorsal portion and uncinate process [10-12]. Figure 1 illustrates the partition separating the ventral and dorsal pancreases. This partition, along the junction of the two parts, is called the EFP [2-7]. The EFP is recognized as the partition separating the ventral and dorsal pancreases. Blood vessels run around the EFP.

Surgical technique with recognition of the EFP

**Figure 2:**
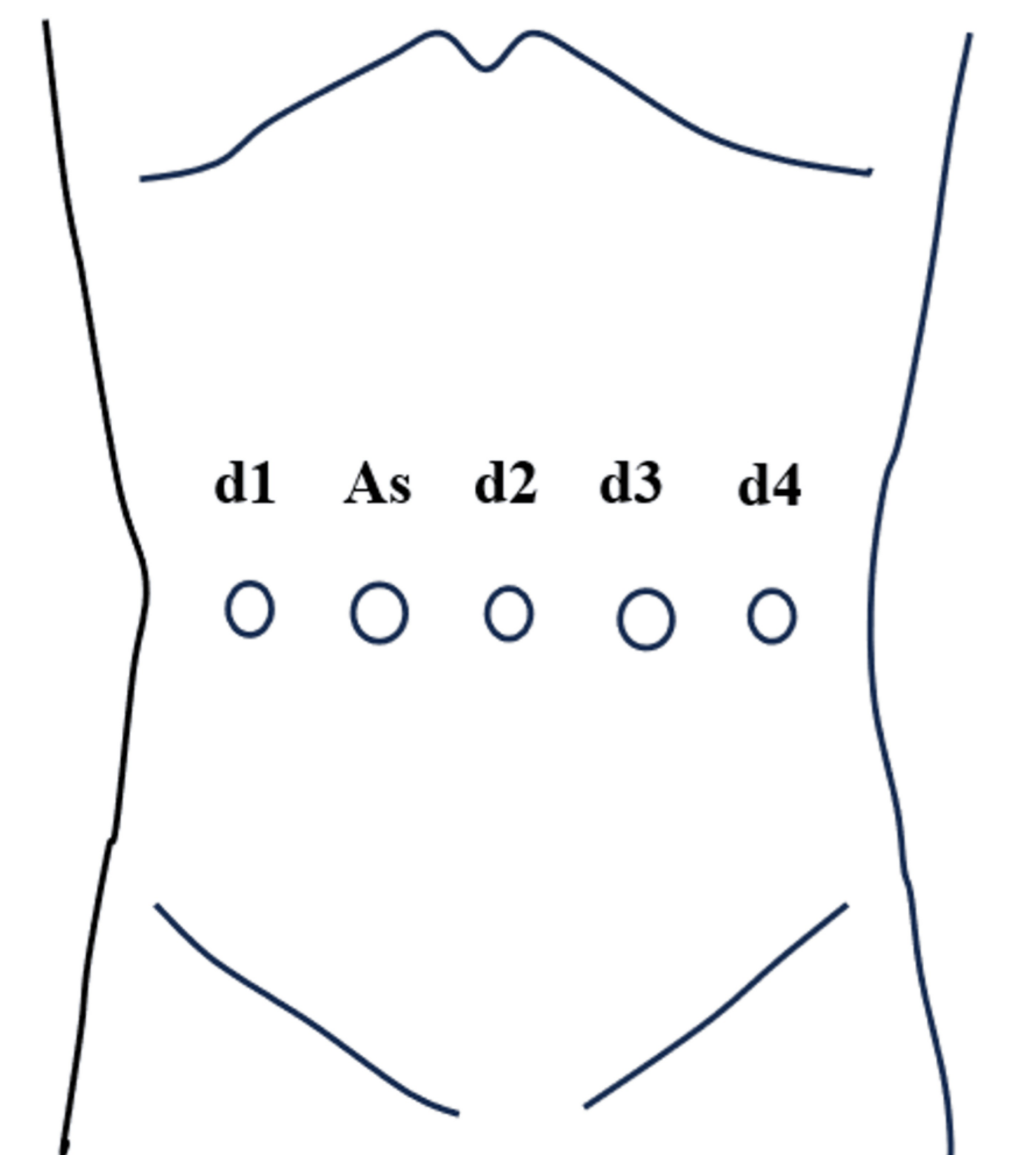
Trocar placement. An 8 mm trocar is placed on the first arm (d1), a 12 mm trocar for the assistant (As), a 12 mm trocar on the second arm at the umbilicus (d2), a 12 mm trocar on the third arm (d3), and an 8 mm trocar on the fourth arm (d4). Image credit: Ippei Yamana.

**Figure 3:**
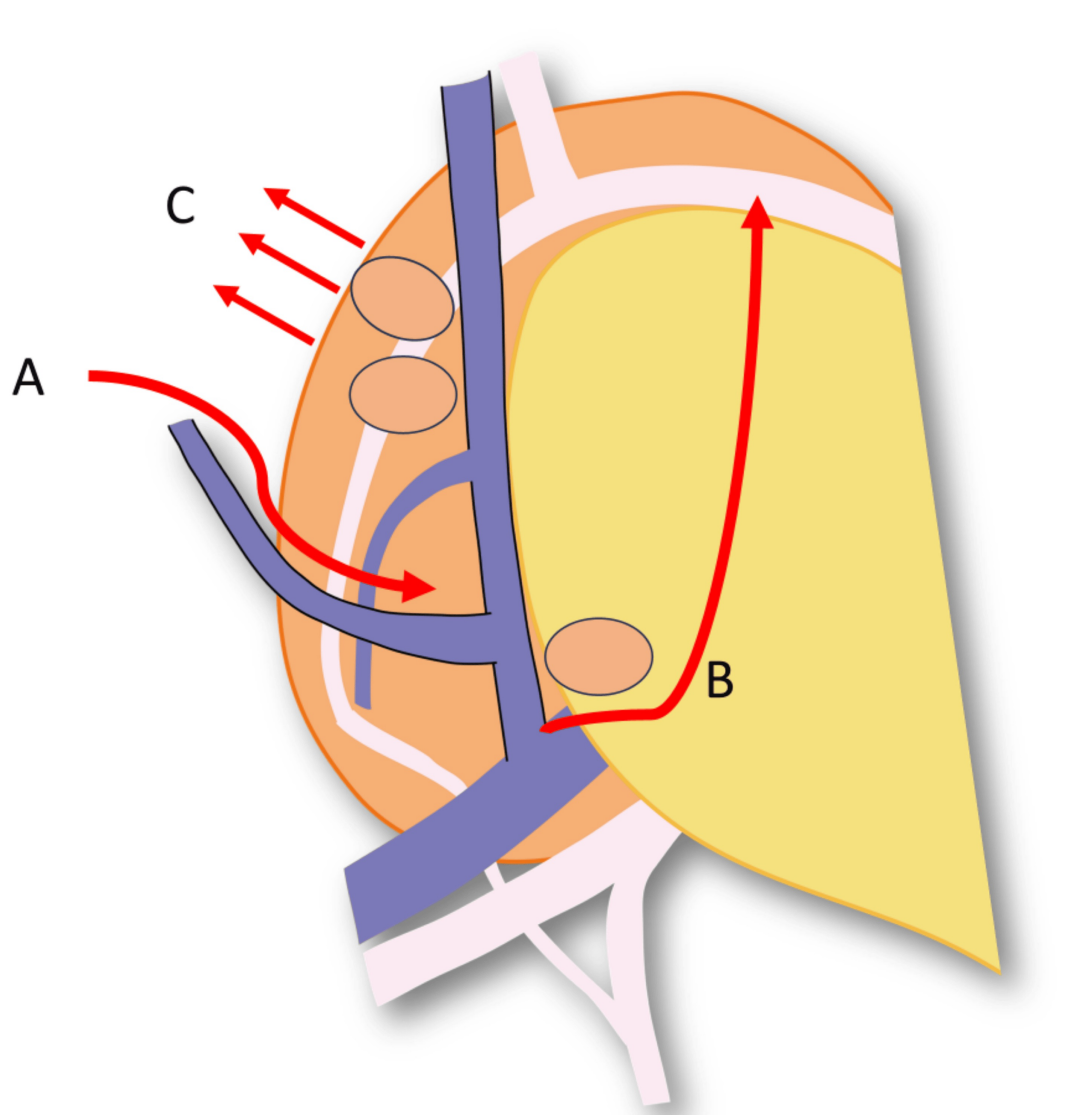
The procedure of infrapyloric lymph node dissection in robotic gastrectomy. (A) The transverse colon mesentery is sufficiently mobilized until the duodenum and the accessory right colic vein are fully visible. (B) The lymphatic chain from the right gastroepiploic vein to the middle colic vein is dissected at the caudal margin. Following the embryological fusion plane (EFP), the right gastroepiploic artery can be naturally identified. (C) The infrapyloric lymph node dissection is conducted along the outermost layer from the right gastroepiploic artery to the anterior superior pancreatic duodenal artery. Image credit: Yuichiro Kawamura.

Gastrectomy is performed using the da Vinci Xi surgical system (Intuitive Surgical, Inc., Sunnyvale, CA, USA). The ports are placed in a straight horizontal line: an 8 mm trocar is placed on the first arm, a 12 mm trocar for the assistant, a 12 mm trocar on the second arm at the umbilicus, a 12 mm trocar on the third arm, and an 8 mm trocar on the fourth arm (Figure 2). The first arm utilizes a fenestrated bipolar forceps, the third arm employs a Maryland bipolar forceps, monopolar scissors, and a vessel sealer, while the fourth arm utilizes a Cadiere forceps. The procedure is shown in Figure 3. Before performing the infrapyloric lymph node dissection, the transverse colon mesentery is sufficiently mobilized until the duodenum and the accessory right colic vein are fully visible. The lymphatic chain from the right gastroepiploic vein to the middle colic vein is dissected at the caudal margin.

**Figure 4:**
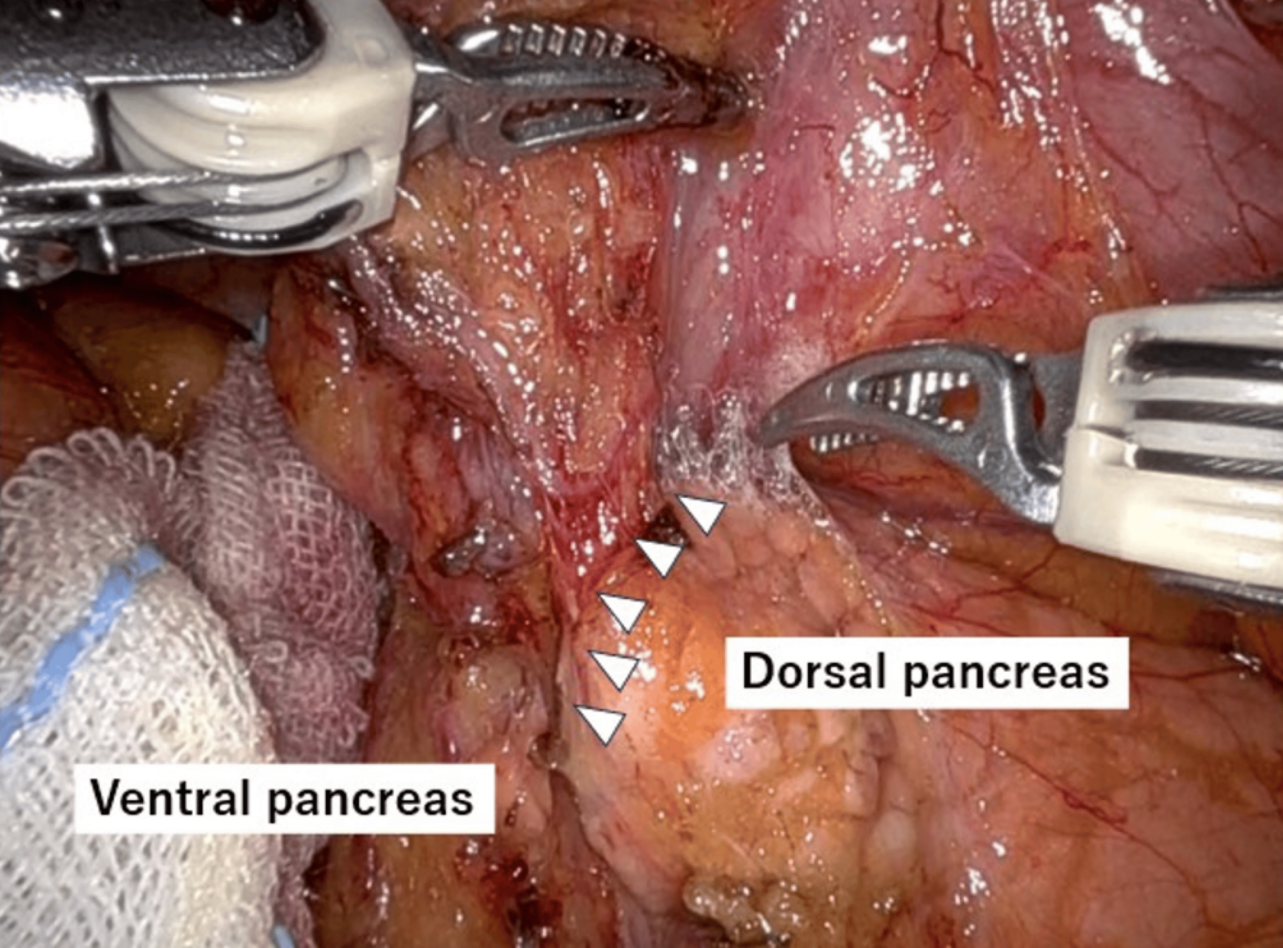
EFP between dorsal pancreas and ventral pancreas during infrapyloric lymph node dissection in robotic gastrectomy. Infrapyloric lymph node dissection is initially performed at the dorsal pancreatic region, which has few vessels and is easy to dissect. Gradually, the EFP becomes naturally recognizable. Arrows: EFP. EFP: Embryological fusion plane.

The dorsal pancreatic surface has few blood vessels and is easy to dissect. On the other hand, the ventral pancreatic surface has many venous branches that flow into the pancreas, and once bleeding occurs, coagulation and hemostasis may lead to pancreatic fistula. Therefore, the infrapyloric lymph node dissection is initially performed at the dorsal pancreatic region, which has few vessels. Gradually, the EFP becomes naturally recognizable (Figure 4). The right gastroepiploic vein is ligated at the periphery of the anterior superior pancreatic duodenal vein. Following the EFP, the right gastroepiploic artery can be naturally identified. The infrapyloric lymph node dissection is conducted along the outermost layer from the right gastroepiploic artery to the anterior superior pancreatic duodenal artery. Finally, the right gastroepiploic and infrapyloric arteries are resected at their roots. The procedure of infrapyloric lymph node dissection in RG with recognition of the EFP is shown in Video 1.

**Video 1:** Infrapyloric lymph node dissection technique in robotic gastrectomy with recognition of embryological fusion plane. Recognizing the EFP during infrapyloric lymph node dissection can help avoid misinterpretation of anatomy and ensure sufficient dissection. EFP: Embryological fusion plane.

## Discussion

The da Vinci Surgical System has made RG possible, enhancing the safety and precision of the procedure. A few studies have documented the safe dissection of the infrapyloric lymph nodes [8,9]. Shinohara H et al. proposed a theoretical concept for mesentery-based D2 gastrectomy by introducing the embryology of the mesogastrium, which reveals its similarity to the mesosigmoid [8].

Fujimoto D et al. suggested that Firefly system-assisted indocyanine green tracer use improves lymph node dissection quality without compromising safety [9]. On the other hand, no studies have been published regarding the dissection of infrapyloric lymph nodes associated with the EFP during RG.

The pancreas is composed of two segments: the dorsal pancreas, which includes the pancreatic neck, body, and tail, as well as the anterior segment of the head, and the ventral pancreas, which includes most of the uncinate process and the posterior segment of the head. Consequently, the head of the pancreas can theoretically be divided into two embryological segments. The plane between the dorsal pancreas and the ventral pancreas is called the EFP [2-7]. The EFP contains few communicating vessels, except for the junction of the dorsal and ventral duct systems [4,13].

In pancreatic surgery, the space between the ventral and dorsal pancreas is referred to as the EFP, which is relatively easy to dissect at sites where resistance is weakened. Alvanos A et al. proposed that landmark-guided dissection of the pancreas, informed by its EFP, facilitates dependable division into morphogenetic compartments [14]. Sakamoto Y et al. suggested that immunohistochemical staining for pancreatic polypeptide effectively differentiates tissue origins and demonstrates the EFP, the border between the dorsal and ventral pancreas [4]. By taking advantage of the EFP, segmental pancreatectomies, such as dorsal pancreatectomy and ventral pancreatectomy, have been performed for benign pancreatic diseases and low-grade tumors in order to preserve pancreatic function [2,6,13,15].

During the dissection of the infrapyloric lymph nodes in RG, we observe a discontinuity in the pancreatic parenchymal surface around the right gastroepiploic artery and the anterior superior pancreaticoduodenal artery. We interpret this discontinuity in the pancreatic parenchyma as the EFP. During RG, we have been able to recognize this EFP more clearly and consistently, thanks to the advantages of 3D magnification. We believe that recognizing the dorsal and ventral pancreas while confirming the EFP can help avoid misinterpretation of anatomy. Consequently, we consider that this technique will assist in preventing bleeding and heat coagulation-related pancreatic fistula.

## Conclusions

We believe that a comprehensive and sufficient dissection of the infrapyloric lymph nodes can be effectively achieved by accurately recognizing both the ventral and dorsal components of the pancreas. Furthermore, it is essential to identify the embryological fusion plane, which serves as a crucial anatomical boundary between these two pancreatic regions. This knowledge not only improves the surgical technique but also lowers the likelihood of complications, ultimately resulting in better patient outcomes.
